# The Indirect Effect of Sleep Quality on Stress-Related Psychosocial Outcomes in Adolescents: An Investigation Across Genders

**DOI:** 10.21203/rs.3.rs-4184541/v1

**Published:** 2024-04-03

**Authors:** Camila Koike, Bridget Nestor, Andreas Baumer, Joe Kossowsky

**Affiliations:** Boston Children’s Hospital, Harvard Medical School; Boston Children’s Hospital, Harvard Medical School; University of Zürich; Boston Children’s Hospital, Harvard Medical School

**Keywords:** Sleep quality, Stress, Adolescents, Indirect effect, Genders, Psychosocial, Psychological

## Abstract

Sleep is foundational for adolescent psychosocial outcomes though often compromised by normative developmental changes and external factors. This cross-sectional study examined sleep quality as a mechanism linking stress and psychosocial outcomes and explored gender differences. Adolescents (N = 246; M_age_=15.8; 46.3% female) completed self-report measures assessing sleep quality and psychosocial outcomes. Structural equation modeling results indicated sleep quality accounted for 78.4% of the total effect of stress on school functioning (*b*=−0.45, *p* < 0.001) and 54.2% of the total effect of stress on pain (*b* = 0.14, *p* = 0.002). A larger indirect effect of sleep quality on school functioning (b=−0.26, *p* = 0.016) emerged for boys than girls, and the effect of sleep quality on pain was significant only for girls (*b* = 0.18, *p* < 0.001, 69.6% of total effect). Sleep quality explained a large proportion of the effect of stress on school functioning and pain. Sleep quality represents a modifiable transdiagnostic pathway that may buffer the effects of stress in adolescence.

## Introduction

Adolescence is a developmental period associated with many biological, psychological, and social changes (APA, 2018). This developmental window also is associated with high levels of stress (APA, 2018). Since the COVID-19 pandemic, adolescent stress levels have increased, with nearly 70% of adolescents reporting higher levels of stress (Mayne et al., 2021). Although high across all adolescents, rates of stress are generally higher for female adolescents (Østerås et al., 2016). Consequently, high levels of stress predict later risk of mental health disorders and have been shown to be associated with numerous negative psychosocial outcomes, including challenges with school functioning peer relationships, and pain (Gazelle & Rubin, 2019; Østerås et al., 2016).

Adolescents also undergo normative changes in sleep, which, similar to stress, have been impacted by the pandemic. Sleep relates to many health and psychosocial domains in adolescence (Dong et al., 2019) and is often quite variable. During the pandemic, difficulties initiating and maintaining sleep increased significantly for adolescents, and meta-analytic work points to a negative impact on sleep from pre- to post-pandemic onset (Correa et al., 2024). Underlying many adolescent sleep difficulties are normative developmental changes including alterations to the sleep/wake homeostatic process and the circadian timing system (Carskadon, 2011). Although the American Academy of Sleep Medicine recommends that adolescents sleep between 8–10 hours each night (Paruthi et al., 2016), most youth report lower sleep duration, poor sleep satisfaction, and poor sleep quality (Dong et al., 2019). Differences related to gender also suggest that female (Marczyk Organek et al., 2015) and nonbinary adolescents (Harry-Hernandez et al., 2020) get less sleep than male adolescents.

Sleep may mediate associations between stressful psychosocial factors in adolescence (Peltz et al., 2019). In addition, poor sleep quality is associated with chronic stress, and increased levels of stress contribute to worse sleep quality (Amaral et al., 2018). Sleep disturbances are also closely related to school-related difficulties as poor sleep quality can lead to increased levels of daytime sleepiness, often associated with worse school performance across genders (Dewald et al., 2010). Insufficient sleep is also associated with poor academic achievement and weakened emotional-behavior regulation (Schmidt & Van der Linden, 2015). Likely related to sleep-related difficulties with emotional-behavioral regulation, ongoing sleep disturbance is also associated with worse interpersonal and social functioning (McGlinchey et al., 2017). Further, sleep is also bidirectionally associated with pain. In adolescents with chronic pain conditions, which typically affect females more than males, poor sleep is associated with increased next-day pain, and increases in pain impact quality of sleep (Bromberg et al., 2012). Less studied, however, is the interplay between sleep and pain intensity in adolescents without diagnosed chronic pain conditions.

The COVID-19 pandemic has had deleterious effects on adolescent well-being, exacerbating an already stress-filled developmental window (Mayne et al., 2021). These effects have had consequences on multiple psychosocial domains, including school functioning, peer functioning, and pain (Kaczynski et al., 2021). For example, in an international study of high school students, adolescents reported decreases in school performance and opportunities to meet friends, even a year after the initial onset of the pandemic (Aluko et al., 2023). Other work has indicated somewhat mixed findings related to pain and the pandemic (Aluko et al., 2023; Kaczynski et al., 2021). For example, although research found decreases in chronic pain prevalence during lockdown, new incidences of chronic pain in youth also increased during this time (Kerekes et al., 2021). No studies to the authors’ knowledge have examined these particular associations in adolescents since lockdown.

The goal of the current cross-sectional study was to examine the effects of sleep quality on associations between stress and school functioning, peer functioning, and pain in a community sample of US adolescents, following the emergency phase of the pandemic. We hypothesized that sleep quality would link the associations between stress and school functioning, peer functioning, and pain. In addition, we investigated binary gender differences for the indirect effect of sleep quality on these psychosocial constructs.

## Methods

### Participants and procedures

Participants were recruited online through the Lookit platform (https://lookit.mit.edu/), which also provided detailed information about the study (e.g., time commitment, compensation, benefits, risks, contact information, etc.). English-speaking adolescents were considered eligible for participation if they were between 14 to 18 years of age and reported no diagnosis of any chronic pain conditions. Adolescent participants and their parents provided informed assent and consent, respectively, online. The survey was delivered via REDCap Platform, and data were captured and stored in REDCap^40^, a secure, HIPAA compliant web-based application. Participants were compensated with a $10 gift card for study participation. This study was approved by the Boston Children’s Hospital IRB.

### Measures

Participants completed the following measures:

### Demographics

Demographic questions assessed participant’s age, race, ethnicity, self-reported gender, grade in school, and caregiver educational attainment.

### Pain

To identify adolescents reporting pain, participants were asked to endorse (yes/no) whether they had experienced any type of aches or pain within the last month (i.e., headache, stomachache, limb pain, etc.). For participants who endorsed this item, the **Numerical Rating Scale** (von Baeyer et al., 2009) was used to assess average pain intensity on a 0–10 scale (0 = *no pain*; 10 = *worst pain imaginable)* and the **Pediatric Pain Screening Tool** (Rau et al., 2021) was used to assess the extent of physical and psychosocial impact of their pain over the past 2 weeks.

### School Functioning

The **SChool REfusal EvaluatioN Scale (SCREEN)** for adolescents (Galle-Tessonneau & Gana, 2019) is a validated self-report questionnaire comprising 18 items assessing school functioning across multiple domains (e.g., anxious anticipation, difficult transition, interpersonal discomfort, and school avoidance). Items were scored on a 5-point scale (*1 = Doesn’t apply to me at all* to *5 = Applies to me completely)*. The total SCREEN score is the sum of all items. Higher scores indicate worse school functioning. Score ranges from 18–90. Cronbach’s alpha for the SCREEN in the current study was 0.95, indicating excellent internal consistency reliability.

### Psychological Stress

The **Patient Reported Outcomes Measurement Information System (PROMIS)** (Cella et al., 2010) **Pediatric Psychological Stress** is a validated self-report assessment of patient outcomes across multiple health domains. The current study used the Short Form version of the PROMIS Psychological Stress measure (Bevans et al., 2018), comprising 8-items. Participants reported the frequency of stress related items in the past seven days on a 5-point Likert scale *(1 = Never to 5 = Almost Always)*. Raw scores were summed and converted into standardized T-scores. The PROMIS scale has demonstrated good reliability and validity in children and adolescents^46^. Higher scores indicate higher psychological stress. Cronbach’s alpha for this measure in the current study was 0.97, suggesting excellent reliability.

### Peer Relationships

The **Patient Reported Outcomes Measurement Information System (PROMIS)** (Cella et al., 2010) **Pediatric Peer Relationship** is a validated self-report assessment (Dewalt et al., 2013). This study used the Short Form version of the PROMIS Peer Relationship, which is comprised of 8 items assessing quality of peer relationships in the past seven days on a 5-point Likert scale *(1 = Never to 5 = Almost Always)*. Raw scores were summed and converted into standardized T-scores. Higher scores indicate better peer relationships. Cronbach’s alpha for this measure in the current study was 0.90, indicating excellent reliability.

### Sleep Quality

The **Adolescent Sleep-Wake Scale (ASWS)** (Shahid et al., 2011) is a validated 10-item self-report questionnaire, which assesses sleep quality in the past month^50^. Three subscales comprise the ASWS: going to bed, falling asleep and reinitiating sleep; and returning to wakefulness. Total scores were obtained by summing all subscales. Participants ranked how often certain sleep statements were true for them in the past month on a 6-point Likert scale *(1 =Never to 6 = Always)*. Higher scores indicate higher sleep quality. The maximum score in each subscale is 5. Cronbach’s alpha for the ASWS in the current study was 0.87, indicating good reliability.

### Data analysis

IBM SPSS Version 28 (IBM SPSS Statistics for Macintosh, Released 2021) was used to conduct descriptive and correlational analyses of the following variables, including their relevant subscales: demographics, sleep quality, psychological stress, peer relationships, school functioning, and pain. For correlational analyses, we reverse coded the SCREEN so that higher scores indicate better school functioning. This was done to ease interpretation of correlations. We also used SPSS to test for group differences between binary gender (male, female) on each variable of interest using t-tests. For t-tests and correlational analyses, we used pairwise deletion to obtain an analysis sample of complete cases. Due to our small sample of non-binary participants, we did not include this sample in the main analyses. Please see supplement for additional information about these participants.

Using the lavaan package for R (Rosseel, 2012), we built a cross-sectional structural equation model (SEM) to assess the extent to which sleep quality accounted for the associations between stress and school functioning, peer relationships, and pain, while controlling for age. Similarly for these analyses, we again reverse coded the SCREEN so that higher scores indicate better school functioning. This was done to ease interpretation of parameter estimates. Confidence intervals and z-test statistics were calculated using bootstrapping, and p-values were adjusted for multiple comparisons using the Benjamini-Hochberg method.

## Results

The total sample consisted of 246 adolescents (mean age=15.83 years, SD=1.04); 93.9% (N=231) provided complete data. See Table 1 for demographic information.

### Psychosocial outcomes

Table 2 provides means and standard deviations (SDs) for each psychosocial variable by gender identity, as well results from t-tests for mean differences between male and female adolescents.

Significant binary gender differences were found between male and female adolescents for the Wakefulness subscale of the ASWS (*t*=−3.09, *p* < 0.01), indicating more wakefulness for males than for females, and for the Anxious Anticipation subscale of the SCREEN (*t*=−2.25, *p*=0.03), indicating more anxious anticipation for males than for females. There were no other significant binary gender differences between male and female adolescents for any other measures (*p*-values > 0.05). No significant binary gender differences emerged for psychological stress, peer relationships, or pain (*p*-values > 0.29).

### Correlation analysis

**Figure 1.**
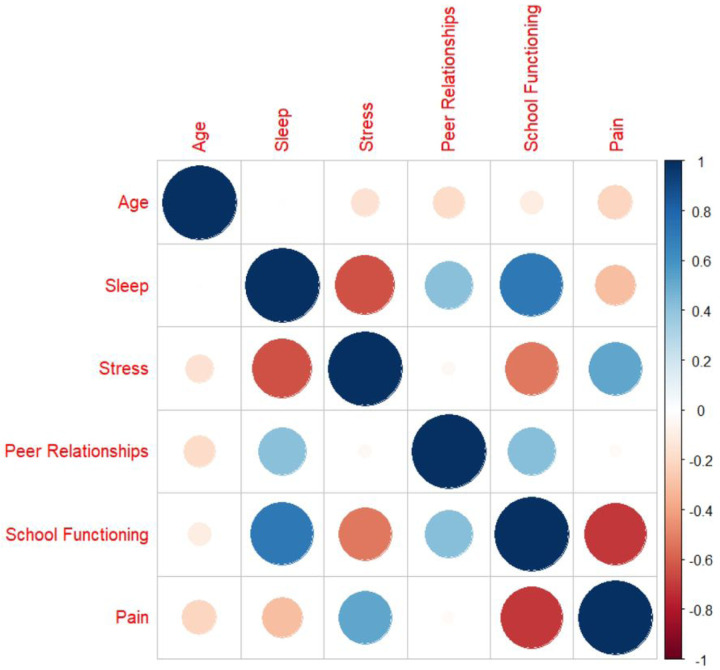
Correlation Heatmap *Note:* Sleep was measured by Adolescent Sleep-Wake Scale (Essner et al., 2015); Stress and Peer Relationships were measured by Patient Reported Outcomes Measurement Information System (Cella et al., 2010) Pediatric Psychological Stress (Bevans et al., 2018) and Pediatric Peer Relationships (Dewalt et al., 2013), respectively; School functioning was measured by the School Refusal Evaluation Scale (SCREEN) (Gallé-Tessonneau & Gana, 2019); pain was measured by the Pediatric Pain Screening Tool (Rau et al., 2021). To ease interpretation of correlations, we reverse coded the SCREEN so that higher scores indicated better school functioning. In doing this, for all measures presented above, higher scores represent more of the measured construct (i.e., higher sleep quality, more stress, better peer relationships, better school functioning, and more pain). Plot was created using *corrplot* (Wei, 2021).

School functioning and overall sleep quality were strongly positively correlated (*r*=0.73, *p*<.001), indicating that better overall sleep quality was related to better school functioning. School functioning was also positively correlated with peer relationships (*r*=0.30, *p*<.001). In addition, peer relationships were negatively correlated with age (*r*=−0.22, *p*<.001). Average pain intensity was negatively correlated with school functioning (*r*=−0.76, *p*<.001), indicating increased pain levels were related to worse school functioning. There was a strong negative correlation between sleep quality and psychological stress, indicating worse sleep quality was associated with more psychological stress (*r*=−0.72, *p*<.001). Moreover, sleep quality was positively correlated with peer relationships (*r*=0.23, *p*<.001). Psychological stress was strongly positively correlated with pain levels (*r*=0.63, *p*<.001) and strongly negatively correlated with total school functioning (*r*=−0.60, *p*<.001). Further, psychological stress was negatively correlated with age (*r*=−0.16, *p*=.014). Psychological stress was not correlated with peer relationships (*r*=0.01, *p*=.916). Figure 1 presents the correlation heatmap. For full correlational output for all variables including their subscales, please see the Supplement.

### Path analyses

**Figure 2:**
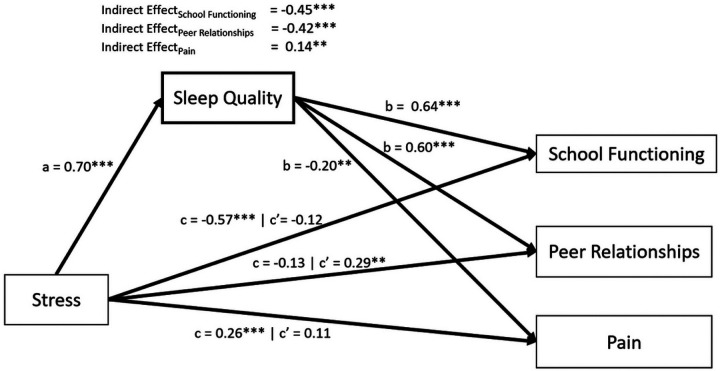
Path Diagram Including All Genders (N=226) *Note:* Sleep was measured by Adolescent Sleep-Wake Scale (Essner et al., 2015); Stress and Peer Relationships were measured by Patient Reported Outcomes Measurement Information System (Cella et al., 2010) Pediatric Psychological Stress (Bevans et al., 2018) and Pediatric Peer Relationships (Dewalt et al., 2013), respectively; School functioning was measured by the School Refusal Evaluation Scale (SCREEN) (Galle-Tessonneau & Gana, 2019); pain was measured by the Pediatric Pain Screening Tool (Rau et al., 2021). To ease interpretation of parameter estimates, we reverse coded the SCREEN in these analyses so that higher scores indicated better school functioning. In doing this, for all measures presented above, higher scores indicate more of the measured construct (e.g., higher sleep quality, more stress, better peer relationships, better school functioning, and more pain). Displayed parameter estimates are standardized and correspond to correlation coefficients. P-values were adjusted for multiple comparisons using Benjamini-Hochberg method. *p-value<0.05; **p-value<0.01; *** *p*-value<0.001.

As reported in our correlation results, associations of stress with sleep, pain, and school functioning were significant. However, peer relationships were not significantly associated with stress, therefore it was excluded from further path analysis (Baron, 1986). The structural equation model is displayed in Figure 2 and results are reported in Table 3. Stress displayed a significant negative effect on school functioning (*b*=0.57, *p*<0.001) and pain (*r*=0.26, *p*<0.001). Sleep quality accounted for 78.4% of the total effect of stress on school functioning (*b*=−0.45, *p*<0.001) and 54.2% of the effect of stress on pain (*b*=0.14, *p*=0.002). For both outcomes, the remaining direct effect of stress was no longer significant once the indirect effect of sleep quality was accounted for (school functioning: *b*=−0.12, *p*=0.07; pain: *b*=0.11, *p*=0.14).

#### Gender Specific Effects

In total, 101 female and 123 male identifying participants were included in the analysis of gender-specific effects. Male participants experienced a larger total effect of stress on school functioning than female participants (*b*=−0.31, *p*=0.002). Sleep quality accounted for 85.0% of the effect of stress for males and 87.7% of the effect for females.

There was no significant difference between male and female participants in the total effect of stress on pain (*b*<0.01, *p*=0.975). However, binary gender did significantly moderate the effect of sleep quality on pain such that the indirect effect of sleep quality on pain was significant for female participants (b=0.18, *p*=0.007; 69.6% of total effect), but not for male participants (*b*=0.08, *p*=0.39). The remaining direct effect of stress on pain was not significant for females (*b*=0.08, *p*=0.317).

## Discussion

The current study investigated the indirect effect of sleep quality on associations between stress and school functioning, peer functioning, and pain in a community sample of adolescents. Consistent with our hypothesis, sleep quality significantly accounted for the effect of stress on school functioning, and to a lesser extent, the effect of stress on pain. Sleep quality did not significantly account for the association between stress and peer functioning. We discuss these results further and suggest their potential implications for future research and clinical practice.

First, our results indicated that sleep quality significantly accounted for the effect of stress on school functioning, explaining 78% of the effect of stress on school functioning across male, female, and non-binary adolescents. Our analyses by binary gender further showed that the indirect effect of sleep quality accounted for 88% of the effect of stress on school functioning for adolescent girls and 85% of the effect for adolescent boys. This finding aligns with prior research showing that insufficient sleep and sleepiness are associated with poorer academic functioning, likely related to the cognitive impact on sleep disturbance for adolescents’ learning, memory, and concentration (Dewald et al., 2010).

Second, our findings revealed that sleep quality significantly accounted for the effect of stress on pain, particularly for adolescent females. This is consistent with prior work in the adolescent pain literature highlighting the negative effects of poor sleep on pain, suggesting that associations between sleep and pain exist for youth, even in the absence of diagnosed pain conditions (Clementi et al., 2020). Contrary to our hypothesis, sleep quality did not link the association between stress and peer functioning in the current sample. Although prior research has indicated negative associations between sleep and interpersonal functioning in adolescents, the current study assessed specifically adolescent social functioning in the context of peers (Sarchiapone et al., 2014). The effect of sleep on interpersonal functioning could possibly be specific to relationships with family and adults, rather than peers. Many studies identifying a link between sleep and social functioning have not reported only on peer functioning, rather more general interpersonal relationships (McGlinchey et al., 2017). Other studies have shown significant associations between sleep disturbances and peer-related problems in adolescence, such as loneliness and victimization or bullying (Kubiszewski et al., 2014). Future research would benefit from clarifying associations between sleep and specific domains of interpersonal functioning.

Our analyses related to gender indicated no significant differences across models for male versus female adolescents. Due to our small sample size, however, we were unable to draw claims about sleep quality for non-binary adolescents. We therefore suggest that future work investigate sleep experiences of gender minority youth as burgeoning evidence indicates high rates of sleep difficulties in both non-binary adults and adolescents (Harry-Hernandez et al., 2020). Better understanding sleep experiences of gender minority youth is critical for bolstering our understanding of adolescent sleep disparities (Marczyk Organek et al., 2015).

Investigating adolescent sleep quality as a mechanism holds promise as a modifiable transdiagnostic pathway toward adolescent health. Indeed, studies show that interventions can improve adolescent sleep through school-based, transdiagnostic, and disorder-specific treatments (Harvey, 2016). Better sleep is associated with positive outcomes, including decreased risk in emotional, cognitive, and social domains (Dong et al., 2019). It is also associated with fewer physical symptoms and lower rates of obesity, mood, and anxiety disorders (Dong et al., 2019). Moreover, sleep is associated positively with memory, language, executive function, overall cognitive development, and physical growth in youth (Tham et al., 2017). Findings from the current study suggest that interventions targeted to improve sleep quality in adolescents may also have downstream effects on their school functioning and pain. Notably, as compared to training adolescents in coping skills for stress-reduction that are often difficult to implement in the moment, sleep-related intervention, such as modifying sleep hygiene behaviors, may represent a relatively accessible approach to psychosocial improvement (Ten Brink et al., 2021).

We suggest that future research build upon the limitations of the current study. First, the current study is cross-sectional, thus limiting our ability to make claims of temporal or causal nature amongst our variables (Maxwell & Cole, 2007). To that end, we suggest future studies examine longitudinally the potential mediating role of sleep quality on associations between stress and psychosocial outcomes over time. Longitudinal investigations can also better parse the often bidirectional association between poor sleep quality and later stress, as well (Ten Brink et al., 2021). Second, our sample consisted of an overwhelming majority of adolescents with binary gender identities. Larger, more gender-diverse samples will be necessary to investigate the sleep experience for non-binary adolescents. Third, our study relied on self-report measures, which are subject to reporter and recall bias (Macchiarola et al., 2022). Specifically, we recommend that future work assessing the mechanistic role of sleep employ objective measures of sleep (e.g., consumer-grade wearable devices, actigraphy watches, etc.). Relatedly, we also suggest future research studies incorporate a more comprehensive assessment of the multidimensional constructs of adolescent sleep health (Meltzer et al., 2021).

In conclusion, the current study tested the cross-sectional mechanistic role of sleep quality on associations between stress and social functioning, peer functioning, and pain in a community sample of adolescents. Our findings highlight how sleep quality may explain a significant proportion of the effect of stress on school functioning, and a smaller portion of the effect of stress on pain. Future research and clinical practice should continue to investigate sleep as a modifiable mechanism underlying adolescent psychosocial outcomes with the potential to buffer the effects of stress.

## Figures and Tables

**Table 1.** Demographic Characteristics of Adolescent Survey Respondents (N = 246)

	Mean (SD)	n (%)
**Age, years**		
	15.83 (1.04)	
**Ethnicity**		
American Indian or Alaska Native		39 (15.9)
Asian		2 (0.8)
Black or African American		71 (28.4)
Hispanic or Latino		4 (1.6)
Native Hawaiian or Other Pacific Islander		3 (1.2)
White		129 (52.4)
Other Race/Mixed Race		3 (1.2)
Prefer not to answer		1 (0.4)
**Gender Identity**		
Female		114 (46.3)
Male		130 9 (52.8)
Non-binary		2 (0.8)
**Primary Caregiver Education Attainment (n = 242)**		
Did not complete high school		2 (0.8) (2)
High school diploma		3 (1.2)
Postsecondary vocational certificate		4 (1.6)
Associate degree		10 (4.1)
Bachelor’s degree		52 (21.1)
Master’s degree		91 (37)
Doctoral degree		80 (32.5)

n, number of cases; SD, standard deviation

**Table 2.** Psychosocial Variables by Gender Identity

	Total (N = 246)		Female (n = 114)		Male (n = 130)		Non-binary (n = 2)		Binary Gender Difference	
	Mean (SD)	N	Mean (SD)	n	Mean (SD)	n	Mean (SD)	n	t	p
Age	15.83 (1.04)	239	15.88 (1.09)	111	15.80 (.993)	126	14.50 (.707)	2	0.54	0.59
School Functioning Total	45.01 (18.94)	232	42.66 (18.76)	114	46.85 (19.09)	130	49.50 (2.12)	2	−1.67	0.10
Anxious Anticipation	12.78 (6.43)	241	11.80 (6.31)	111	13.67 (6.45)	128	10.50 (4.94)	2	−2.25	0.03*
Difficult Transition	11.01 (4.98)	241	10.33 (4.88)	109	11.50 (5.00)	130	16.50 (4.94)	2	−1.83	0.07
Interpersonal Discomfort	10.45 (3.92)	245	9.93 (4.18)	113	10.83 (3.63)	130	15.00 (2.82)	2	−1.78	0.08
School Avoidance	10.41 (5.16)	240	9.44 (5.08)	109	10.77 (5.17)	129	7.50 (4.94)	2	−1.98	0.05
Sleep Quality	3.42 (.778)	245	3.42 (.731)	113	3.46 (.789)	130	1.51 (.314)	2	−.401	0.69
Wakefulness	4.01 (1.14)	245	3.80 (1.40)	113	4.24 (.739)	130	1.00 (00)	2	−3.09	<0.01*
Falling Asleep	3.16 (1.22)	245	3.30 (1.25)	113	3.06 (1.18)	130	2.20 (1.41)	2	1.53	0.13
Going to Bed	3.09 (.888)	245	3.15 (.767)	113	3.07 (.963)	130	1.33 (.471)	2	.690	0.49
Psychological Stress	55.24 (12.05)	246	54.71 (11.55)	114	55.50 (12.45)	130	68.65 (7.99)	2	−.512	0.61
Peer Relationships	46.69 (8.59)	246	47.48 (10.2)	114	46.21 (6.61)	130	33.5 (12.09)	2	1.16	0.24
Pain Intensity	5.58 (2.74)	43	5.05 (2.91)	19	6.08 (2.60)	23	4.00 (0)	1	−1.21	0.23

*Note:* n=number of cases; SD=standard deviation; N=total number of cases; Sleep was measured by Adolescent Sleep-Wake Scale (Essner et al., 2015); Stress and Peer Relationships were measured by Patient Reported Outcomes Measurement Information System (Cella et al., 2010) Pediatric Psychological Stress (Bevans et al., 2018) and Pediatric Peer Relationships (Dewalt et al., 2013), respectively; School functioning was measured by the School Refusal Evaluation Scale (SCREEN) (Gallé-Tessonneau & Gana, 2019); pain was measured by the Pediatric Pain Screening Tool (Rau et al., 2021).

* *p*<.05

**Table 3.** Effects of Stress on School Functioning and Pain: Indirect Effect of Sleep Quality

	Total Effect [95% CI]	Direct Effect [95% CI]	Indirect Effect [95% CI]	% Accounted by Sleep Quality
School Functioning				
Whole Sample	−0.57 [−0.66, −0.46]***	−0.12 [−0.26, 0.02]	−0.45 [−0.56, −0.35]***	78%
Female	−0.38 [−0.54, −0.17]***	−0.05 [−0.23, 0.19]	−0.33 [−0.48, −0.22]***	88%
Male	−0.70 [−0.81, −0.58]***	−0.10 [−0.29, 0.06]	−0.59 [−0.73, −0.44]***	85%
Pain				
Whole Sample	**0.25 [0.12, 0.39]** ***	0.12 [−0.04, 0.29]	**0.14 [0.06, 0.23]** **	54%
Female	**0.26 [0.07, 0.45]** *	0.08 [−0.07, 0.23]	**0.18 [0.08, 0.31]** *	70%
Male	**0.26 [0.08, 0.46]** *	0.18 [−0.09, 0.50]	0.08 [−0.11, 0.25]	30%

*Note:* Confidence Intervals and p-values estimated by bootstrapping. *P*-values are adjusted for multiple comparisons using Benjamini-Hochberg method.

Significant effects are bolded and marked by asterisk:

* *p*-value<0.05

** *p*-value<0.01

*** *p*-value<0.001.

## Data Availability

Deidentified data are available upon request to the senior author (JK).
